# Can targeted metabolomics predict depression recovery? Results from the CO-MED trial

**DOI:** 10.1038/s41398-018-0349-6

**Published:** 2019-01-16

**Authors:** Andrew H. Czysz, Charles South, Bharathi S. Gadad, Erland Arning, Abigail Soyombo, Teodoro Bottiglieri, Madhukar H. Trivedi

**Affiliations:** 1 Department of Psychiatry, University of Texas Southwestern, Dallas, TX 75390 USA; 2 Center of Metabolomics, Institute of Metabolic Disease, Baylor Scott and White Research Institute, 3812 Elm Street, Dallas, TX 75226 USA

## Abstract

Metabolomics is a developing and promising tool for exploring molecular pathways underlying symptoms of depression and predicting depression recovery. The Absolute*IDQ*™ p180 kit was used to investigate whether plasma metabolites (sphingomyelins, lysophosphatidylcholines, phosphatidylcholines, and acylcarnitines) from a subset of participants in the Combining Medications to Enhance Depression Outcomes (CO-MED) trial could act as predictors or biologic correlates of depression recovery. Participants in this trial were assigned to one of three pharmacological treatment arms: escitalopram monotherapy, bupropion-escitalopram combination, or venlafaxine-mirtazapine combination. Plasma was collected at baseline in 159 participants and again 12 weeks later at study exit in 83 of these participants. Metabolite concentrations were measured and combined with clinical and sociodemographic variables using the hierarchical lasso to simultaneously model whether specific metabolites are particularly informative of depressive recovery. Increased baseline concentrations of phosphatidylcholine C38:1 showed poorer outcome based on change in the Quick Inventory of Depressive Symptoms (QIDS). In contrast, an increased ratio of hydroxylated sphingomyelins relative to non-hydroxylated sphingomyelins at baseline and a change from baseline to exit suggested a better reduction of symptoms as measured by QIDS score. All metabolite-based models performed superior to models only using clinical and sociodemographic variables, suggesting that metabolomics may be a valuable tool for predicting antidepressant outcomes.

## Introduction

It has become increasingly clear that depression is heterogeneous in its pathophysiology and treatment outcomes. The development and validation of genetic, proteomic, and/or metabolomic methodologies may be essential in identifying the pathophysiology of disease expression as well as precision medicine for depression. Metabolomics has recently emerged as a particularly valuable field of inquiry in psychiatry because unlike genomics, it captures the dynamic nature of the disease, and unlike proteomics, it measures the final products of complex interactions among numerous proteins, signaling cascades, and cellular environments.^1,2^.

Several groups have studied metabolomic differences in depressed populations relative to healthy controls^3–5^. Additionally, pharmacometabolomic changes following medication treatment have been reported^6–11^, although these studies are limited to a small subset of medications, including sertraline^6,7,10^, citalopram/escitalopram^11^, and ketamine/esketamine^8^. The collective knowledge of metabolomic differences between depressed patients and healthy controls remains difficult to interpret from these studies because of several limitations: (1) most depressed participants were taking medications, and the impact of the various drugs are undefined; (2) several aspects of metabolomics research such as sample preparation, choice of metabolite assays, and statistical analyses were not standardized^1^; and (3) these studies did not account for the heterogeneity of symptomatic presentations despite the fact that metabolomic differences are very likely symptom specific. These limitations have likely led to the lack of uniformity in results between studies. One solution is the employment of standard metabolomics kits with a fixed array of metabolites that have been validated across multiple laboratories^12,13^. Although not without their disadvantages, these kits encourage the study of the same metabolites throughout the metabolomics community and therefore may provide a better understanding of study results as we continue to work toward standardized and well-controlled methods.

In the present study, the Absolute*IDQ*™ p180 kit was used to investigate metabolomic markers as predictors of antidepressant responsiveness from a subset of participants in the Combining Medications to Enhance Depression Outcomes (CO-MED) trial. This platform has been utilized to study metabolomic changes among a wide range of disease states including dementia^14–17^, diabetes^18,19^, cardiovascular disease^20^, and depression, including one study which investigated effects of ketamine or esketamine treatment^8^.

To our knowledge, this is the first paper to describe pharmacometabolomic data from participants exposed to a Serotonin and Norepinephrine Reuptake Inhibitor (venlafaxine), a Norepinephrine-Dopamine Reuptake Inhibitor (bupropion), or the atypical antidepressant, mirtazapine. We utilized an exploratory approach and sought to understand whether any baseline metabolites act as predictors of depression recovery or if changes in metabolite concentration before and after treatment were biologic correlates of recovery. Metabolite values were compared to participants’ clinical and sociodemographic characteristics and Quick Inventory of Depressive Symptomology (QIDS) scores, and then modeled using a hierarchical lasso method to identify the relationships between these variables^21,22^. Using this approach, we were able to identify metabolites that are potentially meaningful biomarkers in subtypes of depression.

## Methods and materials

### Study overview and participants

This study is based on data and plasma samples collected from the CO-MED trial, which recruited 665 treatment-seeking depressed participants who were randomly assigned to one of three treatment arms: escitalopram monotherapy, bupropion-escitalopram combination, and venlafaxine-mirtazapine combination^23^. From the total CO-MED trial population, a subset of participants further consented to provide baseline plasma samples (*n* = 168) and 12-week follow-up plasma samples (*n* = 103). Of the baseline group, 9 did not have an exit QIDS score and were removed from the analysis; of the follow-up group, 20 had metabolite data below the limit of detection, leaving a total of 159 subjects in the baseline group and 83 subjects in the follow-up group available for analysis (all of whom had complete metabolomics and clinical data).

The CO-MED trial used broad inclusion and exclusion criteria to recruit from both psychiatric and primary care clinics, which were chosen to ensure adequate minority representation and a diverse participant group^23^. All study-related procedures or assessments were completed only after obtaining written informed consent from participants. The CO-MED trial was reviewed and approved by the Institutional Review Boards at UT Southwestern Medical Center at Dallas, the University of Pittsburgh Data Coordinating Center, each participating regional center, and all relevant clinical sites. Additionally, the study was monitored by an independent data safety and monitoring board. Further details of the CO-MED trial can be found through clinicaltrials.gov identifier NCT00590863.

### Assessments

At baseline, participants provided clinical and sociodemographic information. These included age, gender, race, Hispanic ethnicity, onset of depression before age 18 years, presence of suicidal ideations at baseline, presence of comorbid medical conditions, presence of anxious features (derived from HRSD_17_ (Hamilton Rating Scale for Depression-17))^23^, melancholic features (derived from clinician-rated IDS (IDS-C))^24^, atypical features (e.g., mood reactivity, leaden paralysis, weight gain or increased appetite, hypersomnia, and interpersonal sensitivity—all derived from IDS-C)^25^, and baseline depression severity.

At baseline and follow-up at 12 weeks, participants completed the 16-item QIDS-Self-Report (QIDS-SR) scale which was the primary depression symptom severity outcome measure in the CO-MED trial. Each QIDS-SR item is scored from 0 to 3. The total score is calculated from nine domains that define a major depressive episode based on responses to each item. The score ranges from 0 to 27, with higher scores indicating greater depression severity^26^. It correlates highly (0.86–0.93) with HRSD_17_^27^. In previous reports, the reported Cronbach’s *α* of QIDS-SR has ranged from 0.86 to 0.87^26^.

### Metabolomic assay

At baseline and week 12, peripheral venous samples were collected in EDTA tubes (purple top) and shipped to the Biologic Core of National Institute of Mental Health Repository and Genomics Resource (NIMH RGR) (RUCDR Infinite Biologics, Piscataway, NJ, USA) following the standard operating procedures set by the NIMH RUCDR. Plasma was extracted on receipt and aliquoted into 12 tubes of 500 µl each and stored at −80 °C. Our group obtained plasma samples from the NIMH RGR, which were transported on dry ice. There were no freeze and thaw cycles for these samples. The levels of metabolite markers were measured by the Center of Metabolomics, Institute of Metabolic Disease, Baylor Scott and White Research Institute (Dallas, TX, USA). All samples were run at the same time, and researchers were blinded to treatment allocation and outcomes.

Plasma samples were analyzed using the targeted metabolomic Absolute*IDQ*™ p180 kit (Biocrates Life Sciences AG, Innsbruck, Austria). This metabolomic platform provides the simultaneous determination of 188 metabolites which includes 40 acylcarnitines, 42 amino acids and biogenic amines (not included in this analysis), 90 glycerophospholipids, 15 sphingolipids, and sum of hexoses. Metabolites were determined either liquid chromatography or flow injection analysis (FIA) coupled to tandem mass spectrometry. Given that this technical difference may bias results toward one class of metabolite and that biogenic amines have more established relevance with depression that is better suited for separate a priori hypothesis testing, they were not included in this analysis. FIA analysis of 40 acylcarnitines, 90 glycerophospholipids, 15 sphingolipids, and sum of hexoses were quantitated by a one-point internal standard with which included (nine isotope-labeled acylcarnitines, one isotope-labeled hexose, one nonlabeled lysophosphatidylcholine (LysoPC), two nonlabeled PCs, one nonlabeled SM, for a total of 14 internal standards). Therefore, quantitation of lipids and a subset of acylcarnitines which did not have analyte-specific internal standards were “semi-quantitative.” MS analysis was carried out on AB Sciex 5500QTRAP (Foster City, CA, USA) equipped with a Shimadzu Nexera ultrahigh pressure liquid chromatograph system (Kyoto, Japan). Reported concentrations were within the quantification range validated for each metabolite. Four P180 kits were used to analyze the entire cohort. Each plate contained three quality control (QC) levels, supplied with the kit, with the following replicates included in each run: low = 1 replicate, intermediate = 4 replicates, and high = 1 replicate. Data were normalized for batch effects by the mean of the intermediate QC 2 across all plates using Met*IDQ*^*TM*^ software package. Concentrations of all metabolites were reported as µmol/L. This targeted metabolomics method has been validated in six testing laboratories to have a median inter-laboratory coefficient of variation of 7.6%, with 85% of metabolites with a median inter-laboratory variation of <20%^13^.

### Statistical analysis

The hierarchical lasso was used to model improvement in depression severity. This method is designed to perform variable selection amongst a large number of potentially correlated main effects and two-way interactions. The baseline sample and the subset of those with baseline and exit plasma were modeled separately. In each case, models were generated with the change in QIDS-SR from baseline to exit as the outcome variable, defined as QIDS-SR at exit minus QIDS-SR at baseline (meaning a negative change indicates a better outcome). The least absolute shrinkage and selection operator, or lasso^21^, is a form of penalized regression that performs variable selection by shrinking some of the regression parameters to 0. The hierarchical lasso^22,28^ is an extension of this technique with more modeling flexibility: it allows all two-way interactions into the model selection process while forcing the model to maintain a hierarchical structure—that is, if a two-way interaction is retained, either one or both of the main effects is also included—allowing for more complex relationships between variables. The final model still retains the form of *y* = ***X****β*, but some of the *β* will be set to 0. Given there are no distributional assumptions involved, standard errors and tests of significance are not available for the regression parameters, nor is it appropriate to report a more traditional metric like adjusted *R*^2^ (because of the bias induced by penalization, looking at only the variance explained is not desirable unless you already have a very good estimate of the bias); however, the final model is still a linear model, and because variable selection is performed, any retained variables are assumed to be influential in the sense that they contribute to the prediction of the change in QIDS-SR score.

A total of six models were generated using the hierarchical lasso. First, two metabolite-free models were generated that were identical, except for the number of included participants (baseline-only cohort and baseline-plus-exit cohort). The remaining four models included clinical/sociodemographic variables alongside metabolites. This included two metabolite scenarios: one using individual metabolites (146 total) and one using ratios/sums of the individual metabolites (24 total). In our metabolite-free, individual metabolite and ratios and sums models, there were 18, 42, and 164 candidate variables and 171, 903, and 13,530 candidate interactions, respectively. While these numbers are large relative to the sample sizes, Lim and Hastie^28^ demonstrate the efficacy of the hierarchical analysis on a molecular dataset with over 100 million potential interactions.

Specific ratios and sums were chosen based on biologic relationships, as recommended in the Absolute*IDQ*™ p180 kit product manual. For each of these, a model was generated using either baseline metabolites from the baseline-only cohort or a calculated percent change of metabolites from the baseline-plus-exit cohort. The calculated percent change was defined as: [metabolite]_exit_ – [metabolite]_baseline_)/[metabolite]_baseline_. All models were fit using the *glinternet* package^28^ in R 3.2.2^29^. Continuous variables were re-scaled to have mean 0 and standard deviation 1, and the penalty term was chosen via 10-fold cross-validation.

Because the sample size was small and we anticipated a number of the metabolites to represent noise with respect to the outcome variable, we were concerned that one instance of cross-validation may give results that were unstable. As such, cross-validation was repeated 20 times (meaning the regression parameter estimates were re-calculated each time) and cross-validation error averaged for model stability. Further, we bootstrapped this entire process a total of 200 times in the hopes of increasing the generalizability of our results in the absence of external validation. For more details on the analysis decisions and methodology used, please see the Supplemental methods.

To determine whether including individual metabolites or ratios and sums of metabolites improved predictive ability above and beyond simply using clinical and demographic features, we tracked the average cross-validated squared error loss (SEL)^28^ across the bootstrapped samples; smaller values indicate better model fit. In addition to comparing the three paradigms (demographic only, ratios/sums of metabolites, individual metabolites), we also include the comparison of a naive model (i.e., using the mean change in QIDS-SR score as our prediction for every participant) to gauge relative efficiency. Lastly, we report the average percentage of times that each variable (or interaction) was retained across the bootstrapped samples.

## Results

Baseline and baseline-to-exit changes in plasma sphingomyelins and phosphatidylcholines after antidepressant treatment were identified as influential in predicting change in QIDS score using the hierarchical lasso to estimate the linear models. Models including metabolites showed improvement over models only including clinical and sociodemographic features.

Clinical and sociodemographic variables of the subgroup of CO-MED participants who provided blood specimens at baseline (*n* = 159) and study exit (*n* = 83) compared to those who provided neither (*n* = 506) (Table 1) did not show statistically significant differences among these clinical and demographic variables, except for age. The mean age was 47.0 years old in the cohort who provided baseline and exit specimen, but 42.1 years old in the rest of the CO-MED participants, with a false discovery adjusted *p* value of <0.001 (Table S1). Therefore, besides age, the plasma subgroups are clinically and demographically representative of the larger CO-MED cohort.

**Table 1:** Clinical and sociodemographic characteristics of CO-MED trial participant subgroups based on plasma collection

Variable	Baseline cohort (*n* = 159)	Baseline-and-exit cohort (*n* = 83)	Non-plasma cohort (*n* = 506)	Non-plasma or baseline-only cohort (*n* = 582)
Age	44.2 (SD = 11.9)	47.0 (SD = 10.8)	42.2 (SD = 13.3)	42.1 (SD = 13.2)
Gender (female)	71% (*n* = 113)	71% (*n* = 59)	67% (*n* = 339)	68% (*n* = 393)
Race (white)	67% (*n* = 106)	70% (*n* = 58)	63% (*n* = 319)	63% (*n* = 367)
Race (black)	25% (*n* = 40)	20% (*n* = 17)	27% (*n* = 136)	27% (*n* = 159)
Race (other)	8% (*n* = 13)	10% (*n* = 8)	10% (*n* = 51)	10% (*n* = 56)
Hispanic	17% (*n* = 27)	18% (*n* = 15)	15% (*n* = 74)	15% (*n* = 86)
BMI	32.0 (SD = 9.2)	32.3 (SD = 8.8)	30.7 (SD = 8.7)	30.8 (SD = 8.8)
Comorbid axis 1 disorders	1.1 (SD = 1.4)	1.0 (SD = 1.3)	1.2 (SD = 1.3)	1.2 (SD = 1.3)
Comorbid axis 3 disorders	1.9 (SD = 1.3)	2.1 (SD = 1.3)	1.8 (SD = 1.3)	1.8 (SD = 1.3)
Baseline statin use	17% (*n* = 27)	22% (*n* = 18)	11% (*n* = 54)	11% (*n* = 63)
Baseline NSAID use	40% (*n* = 64)	39% (*n* = 32)	39% (*n* = 198)	40% (*n* = 230)
Remission	42% (*n* = 67)	48% (*n* = 40)	37% (*n* = 188)	37% (*n* = 215)
Response	58% (*n* = 92)	58% (*n* = 48)	54% (*n* = 275)	58% (*n* = 319)
Escitalopram-placebo treatment	30% (*n* = 47)	25% (*n* = 48)	35% (*n* = 177)	35% (*n* = 203)
Venlafaxine-mirtazapine treatment	37% (*n* = 59)	39% (*n* = 32)	32% (*n* = 161)	32% (*n* = 188)
Bupropion-escitalopram treatment	33% (*n* = 53)	36% (*n* = 30)	33% (*n* = 168)	33% (*n* = 191)
Escitalopram-placebo response	61.7% (*n* = 29)	61.9% (*n* = 13)	55.4% (*n* = 98)	56.2% (*n* = 114)
Venlafaxine-mirtazapine response	55.9% (*n* = 33)	62.5% (*n* = 20)	52.2% (*n* = 84)	51.6% (*n* = 97)
Bupropion-escitalopram response	56.6% (*n* = 30)	50% (*n* = 15)	55.4% (*n* = 93)	56.5% (*n* = 108)
Baseline QIDS	15.5 (SD = 4.1)	14.7 (SD = 3.9)	15.4 (*SD* = 4.3)	15.6 (SD = 4.3)
Exit QIDS	7.3 (SD = 5.3)	6.8 (SD = 4.8)	7.6 (SD = 5.1)	7.6 (SD = 5.2)
Ever attempted suicide	9% (*n* = 14)	12% (*n* = 10)	9% (*n* = 45)	9% (*n* = 49)
Suicidal ideation	16% (*n* = 26)	17% (*n* = 14)	17% (*n* = 84)	17% (*n* = 96)
Abuse before age 18 years (emotional, physical, or sexual),	56% (*n* = 89)	54% (*n* = 45)	48% (*n* = 242)	49% (*n* = 286)
Onset before age 18 years	42% (*n* = 66)	41% (*n* = 34)	46% (*n* = 230)	45% (*n* = 262)
Melancholic features	31% (*n* = 49)	29% (*n* = 24)	35% (*n* = 178)	35% (*n* = 203)
Atypical features	17% (*n* = 27)	17% (*n* = 14)	15% (*n* = 76)	15% (*n* = 89)
Anxious features	72% (*n* = 115)	75% (*n* = 62)	76% (*n* = 382)	75% (*n* = 435)

**Fig. 1:**
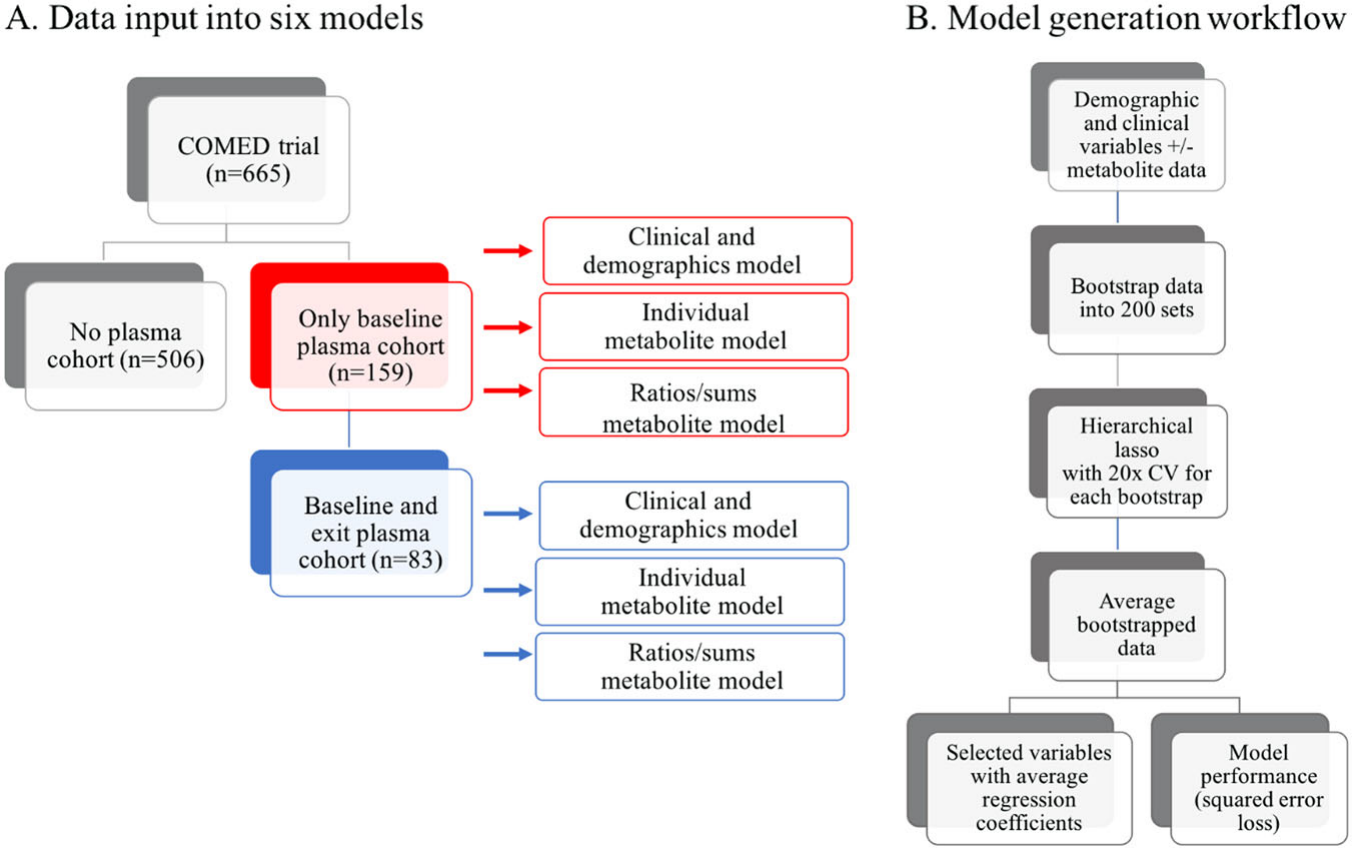
Sub-setting of the CO-MED trial based on baseline and exit plasma cohorts (**a**) and model generation workflow (**b**)

Next, we generated metabolite-free models to determine the relative magnitude of effect of the non-metabolite variables and to validate whether this modeling approach would agree with other literature on these non-metabolite features (Fig. 1). After 200 bootstrap repetitions, each using 20 repeats of 10-fold cross-validation, several variables were retained in most or all of the iterations and there was a stark drop off in the frequency of variable retention after 80% (Figure S2). The presence of comorbid axis 1 and 3 disorders and suicidal behavior were all predictors of smaller changes in QIDS (less improvement). In contrast, higher baseline QIDS, and statin use were predictive of larger changes in QIDS. Treatment arm was also retained, suggesting some minor differences in recovery based on the antidepressant regimen. While this is in contrast with remission rates of the complete CO-MED cohort, it is consistent with the changes in QIDS scores seen across treatment arms. The vast majority of variables, including age, were minimally influential and therefore not included (retained) most of the models. A truncated list of retained clinical and sociodemographic variables in the participant cohort who provided plasma specimens are displayed in Table 2 alongside the average frequency of retention across of the repeated cross-validation and bootstrap replications. The entire list of variables for all analyses is presented in Table S2. Two metabolite-free models were generated: first with the cohort who gave plasma only at baseline, and second with the cohort who also gave blood at study exit. Given the slight difference in clinical features and demographics between baseline and baseline-plus-exit cohorts, there were some differences in variable selection ranking and average regression coefficients between these two analyses (Table 2a, b, respectively). Nonetheless, the average regression coefficients were consistent with respect to magnitude and direction. As an example of how to interpret the information contained in these tables: consider baseline QIDS-SR in Table 2, with an average regression coefficient of −2.75. This means that for every standard deviation increase in QIDS-SR (since the continuous variables were standardized prior to analysis), on average we expect the change in QIDS to increase by 2.75 points. This variable was retained in 100% of the cross-validation repetitions and bootstrap replications, suggesting it is very likely to contain signal. In other words, people who are more depressed at baseline will (on average) lose more points on the QIDS-SR scale (Fig. 1).

**Table 2:** Predictors of change in QIDS using clinical and sociodemographic variables

A. Baseline-only cohort—Demographic variables-only model (*n* = 159)		
Variable/interaction	Average regression coefficient^a^	Frequency of retention in model
Comorbid axis 3 disorders	1.34	100.0
Baseline QIDS	−2.75	100.0
Hispanic	−0.39	99.0
Prior suicide attempt	0.94	98.8
Female gender	−0.13	98.5
Comorbid axis 1 disorders	0.67	98.2
Atypical features	1.02	98.1
**B. Baseline and exit cohort—demographic variables-only model (*n*** = 83)		
Baseline QIDS	−1.61	100.0
Baseline suicidal ideation	1.45	98.4
Comorbid axis 3 disorders	0.62	97.3
Statin user	−0.38	97.3
Comorbid axis 1 disorders	0.60	97.2
Atypical features	0.33	96.2
BMI	0.62	96.1

Models were generated using either the baseline-only cohort (A) or the baseline and exit cohort (B) with baseline clinical and sociodemographic variables. The most influential variables or variable interactions are presented by relative magnitude of effect

^a^Average regression coefficient, as calculated by hierarchical lasso, represents the relative magnitude of effect a variable or variable’s interaction has on predicting change in QIDS. Positive values predictive a smaller change in QIDS, whereas negative values predict a larger change in QIDS (greater decrease)

### Baseline phosphatidylcholine and sphingomyelin metabolites are predictors of change in QIDS

Baseline concentrations of metabolites were incorporated into the hierarchical lasso analysis alongside the clinical/sociodemographic factors. Analyses were conducted both with individual metabolites (Table 3a) and with the ratios and sums of metabolites (Table 3b). PC aa C38:1 was the most influential individual metabolite (average regression coefficient 0.23), suggesting that higher baseline levels predict smaller changes in QIDS. In contrast, the ratio of hydroxysphingomyelin to total sphingomyelin predicted greater changes in QIDS with an average regression coefficient of −0.31. Incorporation of baseline metabolite values into the model did affect the relative influence of the clinical/sociodemographic variables, but most of the same variables were again retained, including comorbid axis 1 and 3 disorders, and baseline QIDS.

**Table 3:** Predictors of change in QIDS using baseline metabolites

A. Baseline-only cohort—individual metabolites model (*n* = 159)		
Variable(s)/interaction	Average regression coefficient^a^	Frequency of retention in model
Baseline QIDS	−2.04	100.0
Comorbid axis 3 disorders	0.61	99.3
NSAID user	0.42	98.8
Anxious features	0.61	96.1
Onset before age 18 years	0.50	94.6
PC aa C38:1	0.23	93.4
LysoPC a C18:2	−0.25	93.3
**B. Baseline-only cohort—ratios and sums of metabolites model (*n*** = 159)		
Comorbid axis 3 disorders	1.13	100.0
Baseline QIDS	−2.57	100.0
Ratio of OH-SM to SM	−0.31	98.6
Escitalopram treatment	−0.44	98.3
Venlafaxine/mirtazapine treatment	0.85	98.3
Escitalopram/bupropion treatment	−0.42	98.3
Female gender	−0.31	98.3

Models were generated using either individual metabolites (A) or metabolite ratios or sums (B), alongside baseline clinical and sociodemographic variables. The most influential variables are presented by relative magnitude of effect

*OH-SM* hydroxysphingomyelin, *SM* sphingomyelin, *LysoPC a* lysophosphatidylcholine, *PC aa* phosphatidylcholine with diacyl residue, *DC-AC* dicarboxy-acylcarnitines, *AC* acylcarnitines

^a^Average regression coefficient, as calculated by hierarchical lasso, represents the relative magnitude of effect a variable or variable’s interaction has on predicting change in QIDS. Positive values predictive a smaller change in QIDS, whereas negative values predict a larger change in QIDS (greater decrease)

### Change in phosphatidylcholine and hydroxysphingomyelin metabolites are biologic correlates of change in QIDS score

The relative changes of various metabolites from baseline to study exit were also modeled alongside demographics using the hierarchical lasso (Table 4a, b). Again, a phosphatidylcholine, this time LysoPC a C20:3, was retained as the most influential individual metabolite. Furthermore, the ratio of hydroxysphingmyelin to total sphingomyelin was again retained as the most influential ratios or sums of metabolite class.

**Table 4:** Predictors of change in QIDS using percent change of metabolites before and after treatment

A. Baseline and exit cohort—percent change individual metabolites model (*n* = 83)		
Variable	Average regression coefficient^a^	Frequency of retention in model
Baseline QIDS	−1.78	100.0
Baseline suicidal ideation	1.10	94.9
Comorbid axis 1 disorders	0.33	93.3
LysoPC a C20:3	0.47	86.1
Onset before age 18 years	0.36	83.5
Hexose	0.25	82.9
Comorbid axis 3 disorders	0.28	82.4
**B. Baseline and exit cohort—percent change ratios and sums of metabolites model (*n*** = 83)		
Baseline QIDS	−1.70	100.0
Comorbid axis 1 disorders	0.48	96.1
Baseline suicidal ideation	1.42	95.3
Atypical features	−0.08	94.9
Statin user	−0.64	94.8
Comorbid axis 3 disorders	0.34	93.6
BMI	0.56	92.9
Ratio of OH-SM to SM	−0.18	92.8

Models were generated using either individual metabolites (A) or metabolite ratios or sums (B), alongside baseline clinical and sociodemographic variables. The most influential variables are presented by relative magnitude of effectn

*PUFA* polyunsaturated fatty acids, *MUFA* monounsaturated fatty acids, *SFA* saturated fatty acids, *OH-SM* hydroxysphingomyelin, SM sphingomyelin

^a^Average regression coefficient, as calculated by hierarchical lasso, represents the relative magnitude of effect a variable or variable’s interaction has on predicting change in QIDS. Positive values predictive a smaller change in QIDS, whereas negative values predict a larger change in QIDS (greater decrease)

### Metabolite and clinical/sociodemographic models outperform naive model

The relative performances of each model are presented in Table 5 using the median SEL. In both cohorts, the demographic and clinical variable-only models vastly outperform the naive models. In all cases adding metabolites further improved the median SEL (and hence model performance). For the baseline-only cohort models, the individual metabolite model had the smallest median error (median SEL 7.5 versus 8.7 for the metabolite-free model). In contrast, when looking at changes in metabolites from baseline to exit, the ratios/sums of metabolite classes generated models with the smallest median error (Table 5b). Distributions of the average SEL values across the bootstrap replicates are shown in Figure S3.

**Table 5:** Relative performance of models based on squared error loss. Lower squared error loss indicates superior model performance

A. Baseline-only cohort models		
Model input	Bootstrap median squared error loss (5%, 95%)	Percent change relative to demographic model
Naive	15.7 (13.1, 19.0)	+80.5%
Individual metabolites	7.1 (4.7, 9.5)	−18.4%
Ratios/sums of metabolites	7.5 (5.3, 10.1)	−13.8%
Demographic	8.7 (6.1, 11.6)	–
**B. Baseline and exit cohort model**		
Naive	11.9 (9.1, 15.5)	+95.1%
Individual metabolites	6.0 ([3.6, 8.5)	−1.6%
Ratios/sums of metabolites	5.5 (3.3, 8.2)	−9.8%
Demographic	6.1 (3.8, 8.8)	–

## Discussion

Using a novel and rigorous statistical approach that simultaneously models hundreds of clinical, sociodemographic, and metabolite variables, this study has identified sphingomyelin and phosphatidylcholine biomarkers that are informative of depression recovery. Increased baseline and changes in the ratio of hydroxysphingomyelin to total sphingomyelin predicted better depression recovery. Baseline levels of the individual metabolites LysoPC a C18:2 was also beneficial, whereas baseline PC aa C38:1 and changes in LysoPC a C20:3 were detrimental. Although clinical features, specifically baseline severity and comorbid axis 3 disorders, were the most predictive model features, all models including metabolites outperformed models excluding them.

To our knowledge, this study is the first metabolomic investigation of depression recovery to use a specialized form of penalized regression such as the hierarchical lasso. Prior metabolomic analyses in depression are limited due to the use of statistical testing that assumes independent observations of metabolites, despite a significant degree of metabolite correlation. In contrast, the lasso observes all variables simultaneously, minimizing false discovery and increasing generalizability of the results. For these reasons, the lasso is an increasingly common tool in genetic research^30,31^, but only recently has begun to see use in metabolomic studies^17,18^.

The validity of the hierarchical lasso for our metabolomic analysis is supported by the results of our metabolite-free models. While metabolomics research on depression is still in infancy, there is a wealth of literature on depression outcomes in relation to clinical and sociodemographic factors. In our two clinical-sociodemographic-only models, retained variables were consistent between both models and with other literature on disease prognosis. High baseline QIDS score predicted an overall greater decrease at exit^32^. The influence of other demographic variables, such as comorbid axis 1 and axis 3 disorders predicting worse QIDS outcomes, is supported by previous literature too^33,34^. Interestingly, while comorbid axis 3 disorders had a negative effect on the outcome, statin use was protective. This is in agreement with a meta-analysis of 9187 patients showing that statin users were 32% less likely to become depressed^35^.

After incorporation of metabolites into the hierarchical lasso model, most of the same influential clinical-sociodemographic variables were retained, as were several metabolites. Although all classes of metabolites examined (sphingomyelins, phosphatidylcholines, and acylcarnitines) were represented in at least some of the models, phosphatidylcholines and sphingomyelins were consistently the most influential. Increased levels of hydroxylated sphingomyelins appeared to be beneficial at baseline, as did changes at exit relative to baseline. Hydroxylated sphingomyelins have been identified in most human tissue, but are the most well studied in the brain and skin^36^. Fatty acid 2-hydroxylase is the primary enzyme responsible for converting SM to OH-SM, and mutations in this gene have been linked to neurologic conditions including leukodystrophy and hereditary spastic paraplegia^36^, but have not yet been associated with psychiatric disorders^37^.

It has also been reported that peripheral sphingomyelinase activity is increased in depression and attenuated by the tricyclic antidepressants imipramine, amitriptyline^38^, and desipramine^39^. Increased plasma levels of the metabolites of sphingomyelin degradation, ceramides, have also been implicated in depression and may play a role in hippocampal apoptosis^40^. Further findings on ceramides in depression are reviewed elsewhere^41^. While sphingomyelinase activity and/or ceramide(s) concentration were not measured in this study, it is plausible that in depression, sphingomyelin degradation preferentially impacts hydroxylated species, which would explain the association with increased hydroxylated species and depression recovery.

The link between plasma sphingomyelins to those in the brain is also undefined, but in neurons and glia, they are critical for signal transduction as key components of lipid rafts. Hydroxylated sphingomyelins are chemically more polar than their non-hydroxylated counterparts, promoting fluidization of lipid rafts^36^. In vitro, fluidization of lipid rafts through cholesterol depletion leads to increased Gα_s_-adenylyl cyclase coupling^42^ and increased cAMP signaling, a key second messenger that regulates brain-derived neurotrophic factor, synaptic plasticity, and neurogenesis. Furthermore, in vitro and in vivo, chronic antidepressant treatment leads to increased Gα_s_-adenylyl cyclase coupling via alterations in lipid rafts^43–45^. In post-mortem human brain tissue, individuals with completed suicide versus other cause of death demonstrated decreased Gα_s_-adenylyl cyclase coupling, again suggesting that lipid raft composition is important in the severity of depression symptoms, including suicidal behavior^46^. In addition, statin use had a protective effect in our models, which as a cholesterol-lowering drug may be exerting similar effects of lipid raft fluidization and therefore augment cAMP signaling.

An increase in total plasma sphingomyelin has also been suggested as a risk factor in cardiovascular disease^47,48^, and given how protective statins were for depression in this cohort, higher levels of total sphingomyelins may explain a common mechanism linking CAD and depression. Alternatively, sphingomyelins may simply be a marker for CAD, and the effects of poor cardiovascular health may be driving depression in these participants. Detailed information on cardiac health was not obtained in this study, and therefore further studies would be necessary to better understand the link between sphingomyelins, heart health, and depression.

### Limitations

While our data suggest the utility of these metabolites as potentially valuable biomarkers for predicting depression treatment outcome, there are several limitations that may affect the results. As this was a secondary analysis from the CO-MED trial, sample size and power for biomarker experiments were not determined a priori. Further, blood draws and plasma extraction were not systematic in collection time or fasting status, and previous studies on this metabolomics platform show higher intraclass correlation coefficients with fasting subjects^12^. While we simultaneously examined metabolites alongside clinical variables that may influence them (such as age, body mass index or comorbid axis 3 disorders), we cannot conclude that these results would translate to a cohort with differences in these variables. Further, we note that averaging the coefficients over a series of cross-validations and bootstrap replications—while hopefully making the results more generalizable—does make them harder to interpret. As is evident in Tables 2–4, some of the larger average effects were not retained in all of the models and some effects that were retained nearly all the time but had relatively small effects.

There are also limitations on the quantitative power of current mass spectroscopy technology, and therefore it would be valuable for future studies to validate these identified variables through other assays. This limitation is further compounded by the relatively small sample size of some clinical subgroups, which may explain why variable interactions were not retained in our models (such as metabolite effects with specific treatment arms). The same Biocrates p180 kit has already been used in a study of ketamine effectiveness, which also showed that a change in hydroxysphingomyelin C22:2 was related to improvement in depression severity score^8^. Although this latter finding was limited by a high false discovery rate, a similar result across different participant cohorts and alternative statistical approaches suggest that further investigation of hydroxysphingomyelins may be fruitful. We chose lasso in part to avoid this issue of high false discovery rate, even though this limits our ability to also use traditional regression models. We decided against performing follow-up statistical testing using more traditional methods after identifying notable metabolites, because performing inference after model selection would treat the identified variables as if they were selected independently of the data, resulting in biased estimates. A lack of a more traditional modeling framework does limit our ability to present traditional statistical approaches such as standard errors (and, as a consequence, *p* values and confidence intervals), but also reduces the risk of misleading or potentially biased results. Future studies should focus on external validation with additional patient populations, more quantitative sphingomyelin assays and a priori hypothesis testing with traditional regression modeling.

In summary, these data offer the first analyses of metabolomics in depression using the hierarchical lasso method and have identified sphingomyelins and specific phosphatidylcholines as predictors and biologic correlates of decreases in depression severity even after controlling for a number of clinical and demographic characteristics.

## Supplementary information

Statistical Methods

Supplemental Table 1
