# Supplementary material for: Can targeted metabolomics predict depression recovery? Results from the CO-MED trial
Source: Transl Psychiatry. 2019 Jan 16;9:11. doi: 10.1038/s41398-018-0349-6 (PMC6341111; doi:10.1038/s41398-018-0349-6)

**Supplemental File – Statistical Methods**

The primary aims of the statistical analysis were to address two questions:

1. Is there evidence that, after accounting for a battery of demographic variables, including metabolites in a predictive model can lead to improved predictions (according to the specifications of this research)?
2. If yes, which metabolites tend to show predictive power, and in what direction?

Penalized regression techniques such as the lasso are popular tools for variable selection; these methods can be thought of as an extension of ordinary least squares (OLS), which estimates regression parameters by minimizing the sum of squared residuals:

$\min_{\beta_{0},\beta} \sum_{i=1}^{N} \left( y_{i}-( \beta_{0}+\beta^{T}\boldsymbol{x}_{\boldsymbol{i}}\boldsymbol{)} \right)^{2}$,

where $y_{i}$ is a continuous outcome of interest for the *i^th^* subject, $\beta_{0}$ is the intercept term, $\beta$ is a $1\times p$ vector of regression parameters, and $\boldsymbol{x}_{\boldsymbol{i}}$ is an $1\times p$ vector of predictor variables for the *i*^th^ subject. The lasso also minimizes the sum of squared residuals, but adds an additional constraints to the sum of the absolute value of the regression coefficients:

$$\min_{\beta_{0},\beta} \sum_{i=1}^{N} \left( y_{i}-( \beta_{0}+\beta^{T}\boldsymbol{x}_{\boldsymbol{i}}\boldsymbol{)} \right)^{2}+\lambda\left\| \beta\right\|_{1}$$

Mathematically, this additional constraint cause shrinkage in the regression parameters – potentially to 0 – thereby removing these parameters from the model entirely (but only those whose estimates have been shrunken to exactly 0). In other words, variable selection is performed. While this shrinkage does cause the resulting estimates to be biased, the variance of the estimate is also reduced as we are optimizing predictive ability. The ultimate goal is to reduce the overall mean-squared error since it is a function of both bias and variance. Nonetheless, we can still interpret the coefficient estimates from the elastic net in the same way that we could with OLS estimates, making this procedure more useful for research where the magnitude and direction of regression effects are of interest.

This research did not utilize the lasso, but one of its further derivatives: the hierarchical lasso (HL). Functionally, it works the same way as the lasso – the sum of squared residuals are minimized with additional constraints imposed, resulting in variable selection. The theoretical development, however, is much more complicated. The primary difference between the lasso and the hierarchical lasso is that the latter allows for all two way interactions as potential candidate variables to be selected. This is beneficial for moderator analyses or for analyses like these where there may be some other interactions of interest (for example, the interaction between nsaid use and a treatment arm).

We decided to use penalized regression over other methods such as random forests, support vector machines, gradient boosting machines, and multivariate adaptive regression splines, for two reasons. First, we wanted effects that could be interpreted in the same manner as those from a traditional linear regression model. Second, our sample size was quite limited. The aforementioned alternatives – while popular as tools for prediction – often require large amounts of data and the effects do not have simple interpretations due to the inclusion of complex, non-linear interactions.

Because of the small sample sizes available for analysis ($n=159$ for the baseline sample and $n=83$ for the percent change sample), we felt it important to build in extra validation steps to reduce the risk of spurious results. Specifically, we utilized repeated cross-validation and bootstrapping. Supplementary Figure 1 documents the flow of the analysis.

**Supplementary Figure 1. Statistical Analysis Flow**

By repeating the modeling process 20 times within each of the 200 bootstrap replicates, we are not able to report a single model; as this is an exploratory analysis on a limited set of data, we feel it more beneficial to the scientific community to report the results as hypothesis generating evidence. As such, this leads to further questions that need addressing:

- Because chance variation is a part of the modeling process, what is the best way to identify “important” variables?
- Once important variables have been identified, how can we quantify the effect?

The chance variation combined with data lacking a strong signal suggested that we should not expect to see identical results within or across bootstrap samples. Supplementary Figure 2 confirms this – it displays an ordered index of the percentage of times each variable combination was retained. For example, in the top left panel, the variable retained most often (though not explicitly labeled on the plot) was baseline severity – it was retained in 100% of the cross-validated repeats in each bootstrap sample. The next largest average retention was comorbid axis 3 disorders, at 99.3%. Note the index values get quite large due to the inclusion of some two-way interactions. Thousands of variables were eligible to be included, but the hierarchical lasso did not always select each possible two-way interaction. Given that thousands of variables *could* have been retained, the plots in general show that the overwhelming majority were not retained and thus did not contain predictive power.

**Supplementary Figure 2.** Ordered Indexes of Percent Retention


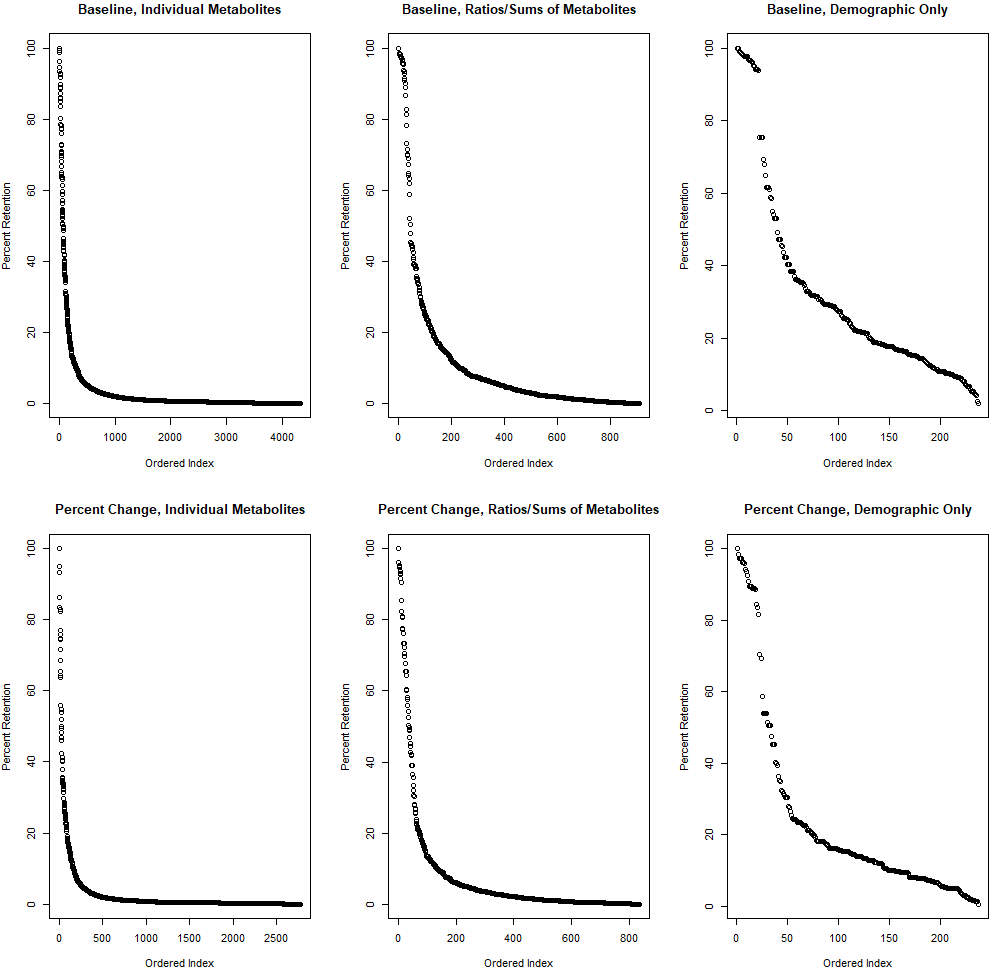


Ideally, the variables with the most predictive power would separate themselves from the rest – functionally, this would look like a drop-off in the plots above. For example, in the baseline, demographic only model there is a drop-off near the 95% retention mark; after this, the next highest retained variable occurs less than 80% of the time. Across the six panels, there is a drop-off near the 80% threshold, so we chose this as our reporting threshold. There are obvious criticisms of this approach: notably, it is not grounded in theory (we could not find any guidance in the literature for this situation) and we may be overlooking meaningful variables. Nonetheless, given that the primary goal of this exploratory analysis was to suggest metabolites with a strong signal, we feel that this approach is reasonable provided that the limitations are clear.

Finally, the heterogeneous model results also made estimating the effects challenging. For example, consider the melancholic depression variable, which was retained on average 88.6% of the time across the bootstrap replicates in the baseline, individual metabolites framework. This means that on average, almost 12% of the time it was not selected – in those situations, the coefficient estimate would be 0. In this case, reporting an average across all replicates will cause the estimates to be biased towards 0 (in the event that the variable is a true predictor), and that bias will likely increase for the less-often-retained variables. However, this does still allow for reporting of the *direction* of the effect – and knowing whether increased levels of metabolites helps/harms depression severity change is still meaningful. So, we have reported the averages noting the limitation that the magnitude of the effects may be dampened.

Finally, in order to gauge the predictive ability of the modeling paradigms, we tracked the squared error loss (SEL) as defined by Lim and Hastie [REF 28 in paper]. As there were 200 bootstrap replications, we could construct distributions of these values. Supplementary Figure 3 below shows these distributions; the median values have been reported in Table 5B.

**Supplementary Figure 3.** Bootstrap Distributions of Average Squared Error Loss


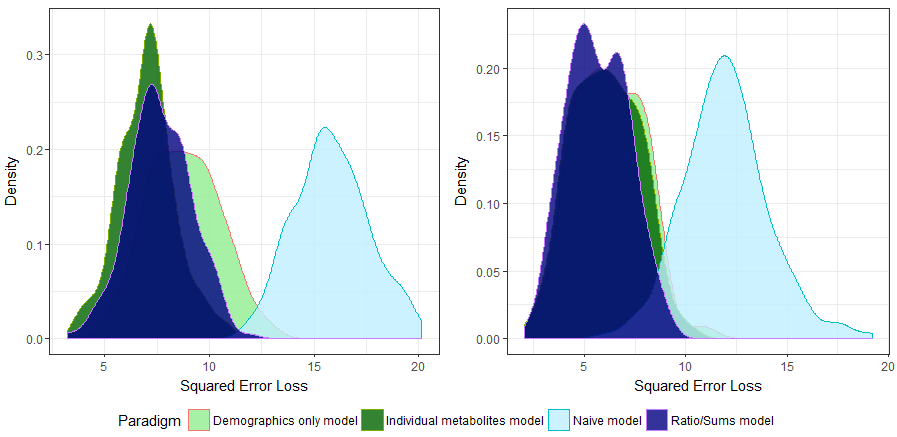

Supplement: Supplementary file 1 — Statistical Methods [file 41398_2018_349_MOESM1_ESM.docx]
