# Supplementary material for: Can targeted metabolomics predict depression recovery? Results from the CO-MED trial
Source: Transl Psychiatry. 2019 Jan 16;9:11. doi: 10.1038/s41398-018-0349-6 (PMC6341111; doi:10.1038/s41398-018-0349-6)
Supplement: Supplementary file 2 — Supplemental Table 1 [file 41398_2018_349_MOESM2_ESM.docx]

**Table S1.** T-test comparisons of clinical and sociodemographic characteristics of CO-MED trial participants sub-groups based on plasma collection.

| **Variable** | **Non-Plasma cohort compared to Baseline Plasma cohort**  **p-value** | **FDR Adjusted** | **Baseline and Exit cohort compared to Baseline Plasma or Non-Plasma Comparison**  **p-value** | **FDR Adjusted** |
| --- | --- | --- | --- | --- |
| Remission | 0.301 | 0.815 | 0.064 | 0.352 |
| Response | 0.493 | 0.815 | 1 | 1 |
| Escitalopram-placebo treatment | 0.355 | 0.815 | 0.204 | 0.561 |
| Venlafaxine-mirtazapine treatment |  |  |  |  |
| Bupropion-escitalopram treatment |  |  |  |  |
| Escitalopram-placebo treatment Response | 0.467^[[1]](#footnote-1)^ | 0.815 | 0.658^[[2]](#footnote-2)^ | 0.852 |
| Venlafaxine-mirtazapine treatment Response |  |  |  |  |
| Bupropion-escitalopram treatment Response |  |  |  |  |
| Baseline QIDS | 0.857 | 0.960 | 0.085 | 0.374 |
| Exit QIDS | 0.556 | 0.815 | 0.192 | 0.561 |
| Gender (Female) | 0.388 | 0.815 | 0.600 | 0.825 |
| Race (White) | 0.691 | 0.893 | 0.418 | 0.784 |
| Race (Black) |  |  |  |  |
| Race (Other) |  |  |  |  |
| Hispanic | 0.551 | 0.815 | 0.536 | 0.801 |
| Baseline Statin Use | 0.047 | 0.638 | 0.008 | 0.088 |
| Baseline NSAID Use | 0.873 | 0.960 | 0.962 | 1 |
| Ever Attempted Suicide | 0.977 | 1 | 0.443 | 0.784 |
| Suicidal Ideation | 1 | 1 | 1 | 1 |
| Abuse before age 18 (emotional, physical, sexual), 1 or more | 0.093 | 0.638 | 0.463 | 0.784 |
| Onset before age 18 | 0.412 | 0.815 | 0.546 | 0.801 |
| Melancholic features | 0.360 | 0.815 | 0.343 | 0.755 |
| Atypical features | 0.638 | 0.877 | 0.834 | 1 |
| Anxious features | 0.438 | 0.815 | 1 | 1 |
| Comorbid axis 1 disorders | 0.731 | 0.893 | 0.329 | 0.755 |
| Comorbid axis 3 disorders | 0.184 | 0.810 | 0.063 | 0.352 |
| BMI | 0.116 | 0.638 | 0.142 | 0.521 |
| Age | 0.078 | 0.638 | <0.001 | 0.006 |

**Table S2.** Complete list of variables retained by hierarchal lasso to predict change in QIDS. MUFA = monounsaturated fatty acids, SFA = saturated fatty acids, PUFA = polyunsaturated fatty acids, OH-SM = hydroxysphingomyelin, SM = sphingomyelin, PC aa = phosphatidylcholine with diacyl residue, PC ae = phosphatidylcholine with acyl-alkyl residue

| **A. Baseline Only Cohort - Demographic Variables Only Model (**$\boldsymbol{n=159}$**)** | | | |
| --- | --- | --- | --- |
| **Variable / interaction** | **Average Regression Coefficient** | | **Frequency of  retention  in model** |
| Comorbid axis 3 disorders | 1.34 | | 100.0 |
| Baseline QIDS | -2.75 | | 100.0 |
| Hispanic | -0.39 | | 99.0 |
| Prior suicide attempt | 0.94 | | 98.8 |
| Female gender | -0.13 | | 98.5 |
| Comorbid axis 1 disorders | 0.67 | | 98.2 |
| Atypical features | 1.02 | | 98.1 |
| Caucasian race | -0.57 | | 97.8 |
| African-American race | 0.64 | | 97.8 |
| Other race | -0.34 | | 97.8 |
| Statin user | -1.08 | | 97.8 |
| NSAID user | 0.58 | | 97.0 |
| Melancholic features | 0.96 | | 96.6 |
| BMI | 0.03 | | 96.5 |
| Baseline suicidal ideation | 1.79 | | 96.4 |
| Onset before age 18 | 0.22 | | 96.0 |
| Early life trauma | 0.21 | | 95.2 |
| Current age | 0.05 | | 95.0 |
| Escitalopram treatment | -0.61 | | 94.2 |
| Venlafaxine/mirtazapine treatment | 0.82 | | 94.2 |
| Escitalopram/bupropion treatment | -0.27 | | 94.2 |
| Anxious features | 0.54 | | 93.9 |
| **B. Baseline And Exit Cohort - Demographic Variables Only Model (**$\boldsymbol{n=83}$**)** | | | |
| Baseline QIDS | -1.61 | | 100.0 |
| Baseline suicidal ideation | 1.45 | | 98.4 |
| Comorbid axis 3 disorders | 0.62 | | 97.3 |
| Statin user | -0.38 | | 97.3 |
| Comorbid axis 1 disorders | 0.60 | | 97.2 |
| Atypical features | 0.33 | | 96.2 |
| BMI | 0.62 | | 96.1 |
| Female gender | 0.36 | | 95.9 |
| Early life trauma | 0.01 | | 94.1 |
| Melancholic features | 1.42 | | 93.5 |
| Onset before age 18 | 0.48 | | 92.5 |
| Current age | -0.04 | | 90.9 |
| Escitalopram treatment | -0.54 | | 89.4 |
| Venlafaxine/mirtazapine treatment | 0.13 | | 89.4 |
| Escitalopram/bupropion treatment | 0.35 | | 89.4 |
| Caucasian race | -0.31 | | 88.8 |
| African-american race | 0.20 | | 88.8 |
| Other race | 0.05 | | 88.8 |
| Prior suicide attempt | 0.80 | | 88.7 |
| Anxious features | 0.09 | | 84.4 |
| NSAID user | -0.27 | | 83.6 |
| Hispanic | -0.08 | | 81.5 |
| **C. Baseline Only Cohort - Individual Metabolites Model (**$\boldsymbol{n=159}$**)** | | | |
| Baseline QIDS | -2.04 | | 100.0 |
| Comorbid axis 3 disorders | 0.61 | | 99.3 |
| NSAID user | 0.42 | | 98.8 |
| Anxious features | 0.61 | | 96.1 |
| Onset before age 18 | 0.50 | | 94.6 |
| PC aa C38:1 | 0.23 | | 93.4 |
| LysoPC a C18:2 | -0.25 | | 93.3 |
| Early life trauma | 0.06 | | 93.3 |
| Hispanic | -0.48 | | 92.8 |
| PC aa C30:2 | 0.08 | | 92.6 |
| Comorbid axis 1 disorders | 0.34 | | 92.5 |
| Female gender | -0.39 | | 91.8 |
| Escitalopram treatment | -0.28 | | 91.7 |
| Venlafaxine/mirtazapine treatment | 0.38 | | 91.7 |
| Escitalopram/bupropion treatment | -0.10 | | 91.7 |
| Acetylcarnitine | -0.04 | | 89.7 |
| Atypical features | 0.43 | | 89.1 |
| Melancholic features | 0.29 | | 88.6 |
| Prior suicide attempt | 0.40 | | 87.3 |
| PC ae C42:0 | 0.08 | | 86.2 |
| PC ae C38:1 | 0.07 | | 86.1 |
| BMI | 0.09 | | 85.9 |
| Statin user | -0.43 | | 85.1 |
| Butenylcarnitine | -0.06 | | 83.7 |
| PC ae C44:4 | -0.01 | | 80.2 |
| **D. Baseline Only Cohort - Ratios and Sums Metabolites Model (**$\boldsymbol{n=159}$**)** | | | |
| Comorbid axis 3 disorders | | 1.13 | 100.0 |
| Baseline QIDS | | -2.57 | 100.0 |
| Ratio OH-SM to SM | | -0.31 | 98.6 |
| Escitalopram treatment | | -0.44 | 98.3 |
| Venlafaxine/mirtazapine treatment | | 0.85 | 98.3 |
| Escitalopram/bupropion treatment | | -0.42 | 98.3 |
| Female gender | | -0.31 | 98.3 |
| Anxious features | | 0.65 | 98.2 |
| Melancholic features | | 0.50 | 97.8 |
| Hispanic | | -0.32 | 97.7 |
| NSAID user | | 0.42 | 97.4 |
| Ratio of PUFA to MUFA | | -0.32 | 97.1 |
| Atypical features | | 0.73 | 97.0 |
| Comorbid axis 1 disorders | | 0.49 | 96.7 |
| BMI | | -0.01 | 96.6 |
| Statin user | | -0.88 | 95.7 |
| Caucasian race | | -0.44 | 95.6 |
| African-american race | | 0.49 | 95.6 |
| Other race | | -0.12 | 95.6 |
| Onset before age 18 | | 0.78 | 95.3 |
| Ratio of DC-AC to AC | | 0.33 | 93.8 |
| Prior suicide attempt | | 0.78 | 93.5 |
| Current age | | 0.06 | 92.7 |
| Ratio of MUFA to SFA | | 0.21 | 91.6 |
| Early life trauma | | 0.22 | 90.8 |
| Ratio of PUFA to SFA | | 0.05 | 90.0 |
| Ratio of OH-AC to AC | | 0.00 | 88.8 |
| Baseline suicidal ideation | | 1.07 | 86.8 |
| Ratio of long chain acylcarnitines to free carnitine | | -0.12 | 82.7 |
| Ratio of SM to PC | | 0.08 | 81.4 |
| **E. Baseline And Exit Cohort - Percent Change Individual Metabolites Model (**$\boldsymbol{n=83}$**)** | | | |
| Baseline QIDS | | -1.78 | 100.0 |
| Baseline suicidal ideation | | 1.10 | 94.9 |
| Comorbid axis 1 disorders | | 0.33 | 93.3 |
| LysoPC a C20:3 | | 0.47 | 86.1 |
| Onset before age 18 | | 0.36 | 83.5 |
| Hexose | | 0.25 | 82.9 |
| Comorbid axis 3 disorders | | 0.28 | 82.4 |
| **F. Baseline And Exit Cohort - Percent Change Ratios and Sums Metabolites Model (**$\boldsymbol{n=83}$**)** | | | |
| Baseline QIDS | | -1.70 | 100.0 |
| Comorbid axis 1 disorders | | 0.48 | 96.1 |
| Baseline suicidal ideation | | 1.42 | 95.3 |
| Atypical features | | -0.08 | 94.9 |
| Statin user | | -0.64 | 94.8 |
| Comorbid axis 3 disorders | | 0.34 | 93.6 |
| BMI | | 0.56 | 92.9 |
| Ratio of OH-SM to SM | | -0.18 | 92.8 |
| Early life trauma | | -0.28 | 91.6 |
| Onset before age 18 | | 0.52 | 90.5 |
| Ratio of lysoPC to PC | | 0.20 | 85.5 |
| Female gender | | -0.05 | 82.4 |
| Ratio of PUFA to MUFA | | 0.05 | 81.1 |
| Ratio of PUFA to SFA | | 0.20 | 80.8 |

1. P-value from a Mantel–Haenszel test for an association between samples and response across drug groups [↑](#footnote-ref-1)
2. P-value from a Mantel–Haenszel test for an association between samples and response across drug groups [↑](#footnote-ref-2)
